# Tridimensional assessment of the dental follicle dimensions of impacted mandibular third molars using cone-beam CT

**DOI:** 10.4317/jced.54310

**Published:** 2018-08-01

**Authors:** Marlene Barroso, Luis-Ernesto Arriola-Guillén, Yalil-Augusto Rodríguez-Cárdenas, Gustavo-Armando Ruíz-Mora, Maria-Eugenia Guerrero, Carlos Flores-Mir

**Affiliations:** 1 Division Oral and Maxillofacial Radiology, School of Dentistry, Universidad Científica Sur, Lima, Perú; 2 Division of Orthodontics, School of Dentistry, Universidad Científica del Sur, Lima, Perú; 3 Division of Oral and Maxillofacial Radiology, Faculty of Dentistry, Universidad Nacional de Colombia, Bogotá, Colombia; 4 Division of Orthodontics, Faculty of Dentistry, Universidad Nacional de Colombia, Bogotá, Colombia; 5 Department of Dentistry, Faculty of Medicine and Dentistry, University of Alberta, Edmonton, Canada

## Abstract

**Background:**

The present study was performed to compare follicle dimensions of impacted mandibular third molar (IMTM) with different impaction angulations using cone beam computed tomography (CBCT).

**Material and Methods:**

Forty-nine individuals with IMTM (24 male, 25 female) were selected. Their age range was 25-55 years. The sample was divided into three IMTM groups either vertical (n=16), mesioangular (n=18) or horizontal (n=15) position based on Winter’s classification (the angle between the longitudinal axis of the second and third molars). Follicular spaces (FS) from available CBCT imaging were measured from the midpoint of the teeth’s crown in several dimensions (mesial, distal, occlusal, apical, vestibular and lingual) in axial, sagittal and coronal planes. An ANOVA, T-student, Kruskal–Wallis and Mann-Whitney U tests were used.

**Results:**

A comparison of the mesial FS for all groups revealed significant differences (*p*<0.001). Significant difference was also found for vestibular FS between the vertical and mesioangular IMTM groups (*p*=0.04). Buccolingual FS for all groups revealed no significant differences (*p*=0.074), whereas significant difference was found for the vertical and horizontal IMTM groups (*p*=0.02). No significant statistical differences were found for occlusal (*p*=0.54), apical (*p*=0.06), and lingual (*p*=0.64) FS.

**Conclusions:**

In this sample IMTM follicles have different dimensions according to their degree of angulation. Mesioagulated and horizontally positioned IMTMs seems to consistently have some increased FS dimensions (mesial and vestibular aspects).

**Key words:**Dental follicle, impacted tooth, third molar, cone-beam computed tomography.

## Introduction

An impacted tooth can be defined as a tooth that is unable to erupt into its position within its expected eruption time because of malposition or lack of space (1). Third molars are the most frequently impacted teeth (2,3). They do normally erupt sometime between 18 and 25 years of age (4). The prevalence of third molar impaction ranges from 16.7% to 68.6% (5-7). Most studies have reported a higher frequency in females than males (8-11).

The dental follicle (DF) around impacted teeth has potential to develop pathological conditions (12,13). Radiographically, the DF appears as a thin pericoronal radiolucency considered normal when it is less than 3 mm thick (12-18). According to the recommendations of National Institute of Health (NIH) both impacted and erupted mandibular third molars with evidence of follicular enlargement should be considered for extraction and the associated soft tissue submitted for microscopic examination (19). It has to be noted that there has not been an internationally accepted consensus on radiographic criteria to differentiate between normal and abnormal conditions of follicular tissue around impacted third molars (14).

A significant part of the problem is related to different magnification rates and spatial tissue overlap expected in either analog or digital two-dimensional radiography (15). It has been speculated that CBCT imaging could be more accurate than conventional two-dimensional radiographic methods in displaying third molar morphology and its related anatomical structures (17). However, CBCT imaging must be properly justified for each patient and should only be requested when there is a potential to provide new information that can impact clinical management decisions not offered by conventional radiography (15,18).

The most frequently observed third molar impaction is a mesioangular position, with a relative frequency of 50 %. There is some controversy regarding how the impaction position may be associated to an increased pathological risk. Some claimed that an horizontally position tooth tends to be the most affected by pathological changes (20), while others maintained that there is no evidence that intraosseous position of a impacted mandibular third molar (IMTM) could lead to an increased risk of developing a cyst or tumor (21-24). It has also been suggested that if a more accurate assessment of its angulation could be provided then this information could serve as an additional tool to justify treatment management decisions (21).

Neither the thickness of the follicle nor the associated three-dimensional positions of the mandibular third molars have been reported yet through CBCT imaging on cases with strong suggestion of impaction. Therefore, the purpose of this study was to compare follicle dimensions of impacted mandibular third molar with different impaction angulations (mesioangular, vertical or horizontal positions) through cone beam computed tomography (CBCT). We tried to refuse the null hypothesis that there are not differences in the follicle dimension between different angulations of impacted third molar.

## Material and Methods

-Sample characteristics

This cross-sectional study was approved by the ethical committee of the School of Dentistry, Científica del Sur University-Lima, Perú, (N° 000227). This study included 49 CBCTs (24 male, 25 female) with impacted mandibular third molar (IMTM) previously taken for reasons not related to this study at CDI Diagnostic Imaging Center in Lima, Perú. Their age range was 25-55 years (mean age 28.6 years old). The sample was divided into three IMTM groups either vertical (n=16), mesioangular (n=18) or horizontal (n=15) position based on Winter’s classification (the angle between the longitudinal axis of the second and third molars). The sample size calculation was determined considering a mean difference of 1.5 mm in the mesial dimensions of the follicular spaces as a clinically relevant difference between impacted third molar with vertical and horizontal angulations. A standard deviation of 1mm was considered (obtained from a preliminary pilot study) with a two-sided signiﬁcance level of 0.05 and a power of 80%. Although a minimum of 6 IMTM was required, a minimum of 15 IMTM were available.

The inclusion criteria consisted on the following.

**Figure 1:**
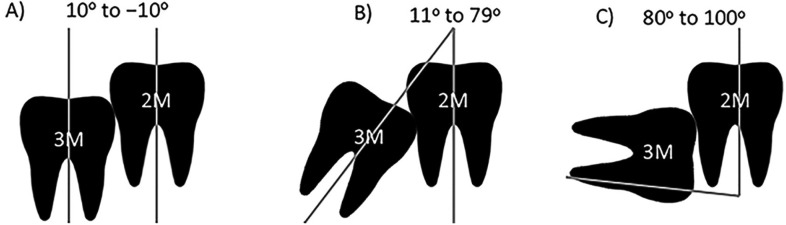
IMTM in vertical, mesioangular or horizontal position. A) Vertical impaction: 10o to −10o; B) Mesioangular impaction: 11o−79o; C) Horizontal impaction: 80o-100o.

1) CBCT scans taken with the same machine under the same parameters from January 2014 to December 2014.

2) Participants aged between 25 and 55 years.

3) IMTM in either vertical, mesioangular or horizontal position based on Winter’s classification (21) (the angle between the longitudinal axis of the second and third molars is categorized as follows: Vertical impaction: 10oto −10o; mesioangular impaction: 11o−79o; horizontal impaction: 80o-100o) (Fig. 1);

4) IMTM class C based on Pell y Gregory’s classification (the relation of the cementoenamel junction (CEJ) of the third molar with the bone level is categorized as follows: Level A: Not buried in bone; level B: Partially buried in bone if any part of CEJ was lower than bone level; level C: Completely buried in bone).

5) IMTM with complete root formation.

Exclusion criteria were as follows.

1) IMTM class A and class B (Pell y Gregory’s classification).

2) Presence of any pathological and developmental conditions in the area surrounding the IMTM (i.e., tumors, cysts, fractures, or malformations).

3) Obvious alterations in size and in shape of IMTM, and 

4) Presence of any artifacts or blurring affecting image quality around the are of interest.

-Cone beam computed tomography examination

Imaging was performed with a Picasso Master 3D (Vatech, E-WOO Technology Co, Ltd, Republic of Korea) CBCT machine. Device settings were set at 8 mA and 90 kV. Each ﬁeld of view (FOV) mode was 20 cm x 19 cm, and with an isotropic voxel size of 0.4 mm.

**Figure 2:**
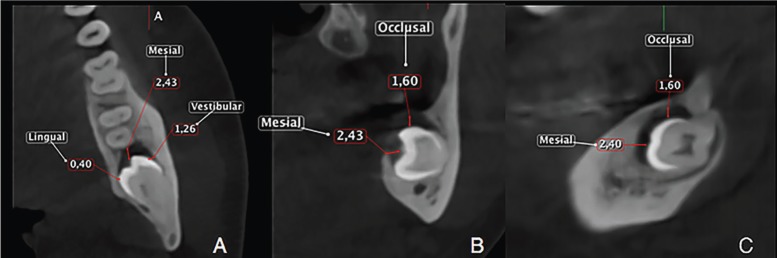
FS measurements performed on different CBCT sections. A. Axial view showing vestibular, mesial and lingual FS measurements on a horizontal impacted third molar B. Vestibular, occlusal and lingual FS measurements performed on a coronal view C. Sagittal view of the FS occlusal measurement.

Participants were in centric occlusion (maximum intercuspidation) during CBCT imaging. The CBCT volumetric data sets (one for each mandibular third molar region) were reconstructed in three planes: axial, sagittal and coronal. The data sets were viewed using EZ Implant 3D software v.1.5 (Vatech, E-WOO Technology Co, Ltd, Republic of Korea). All images were assessed in a dark room on one monitor (S19C150 Samsung 18.5 inch flat panel liquid-crystal display monitor - SAMSUNG, South Korean) set at a resolution of 1366 x 768 pixels. The subject’s head scan was positioned based on the Frankfurt plane (Po-Or). Follicular spaces (FS) were measured from the midpoint of the teeth’s crown in several dimensions (mesial, distal, occlusal, apical, vestibular and lingual) in axial, sagittal and coronal planes (Fig. 2). All patients were informed in advance that the scans might be anonymously used for research reasons later and their consent was obtained. An intra-examiner calibration procedure consisted on the primary investigator measuring 5 pairs of CBCT images 2 times after one week. Intra-examiner reliability was assessed with the intraclass correlation coefficient (ICC).

-Statistical evaluation

Statistical analyses were performed by transferring all the data on Microsoft Excel 2011 software (Microsoft Corporation) and the Statistical Package for the Social Sciences software SPSS v.21 for MAC (IBM SPSS, Armonk, NY: IBM Corp.) was used. Normal distribution was confirmed by Shapiro-Wilk tests in some groups (coronal). A one-way analysis of variance (ANOVA) test and several t-tests were performed to determine whether there were differences in diameter of FS in the six measured dimensions and according to IMTM angulation. The equivalent non-parametric Kruskal-Wallis test and Mann-Whitney U-test was used when normality was not satisfied in some groups (vestibular, lingual, mesial, distal, apical). Statistical significance was set at *p*< 0.05 for all tests.

## Results

Intra-examiner reliability gave a result greater than 0.90 for all measurements (confidence intervals between 0.900 - 0.998).

Descriptive statistics were used to summarize the sample characteristics according to IMTM angulation, sex and age. A comparison of the mesial FS for all groups revealed significant differences (*p*<0.001) ( Table 1). Moreover, significant difference was found for vestibular FS between the vertical and mesioangular IMTM groups (*p*=0.04) ( Table 2). However, buccolingual FS for all groups revealed no significant differences (*p* =0.074), whereas significant difference was found for the vertical and horizontal IMTM groups (*p*=0.02) ( Table 3). No significant statistical differences was found for occlusal (*p*=0.54), apical (*p*=0.06), and lingual (*p*=0.64) FS ( Table 4).

**Table 1:**
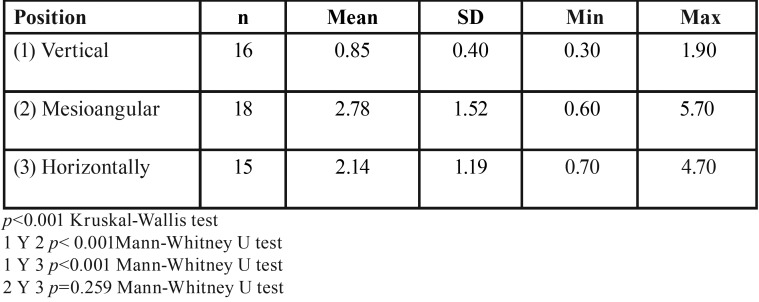
Comparison of the mesial dimensions of the follicular spaces depending on the molar position.

**Table 2:**
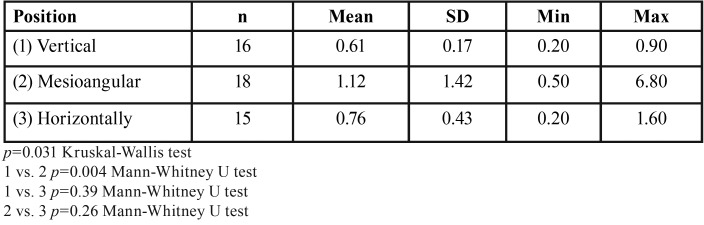
Comparison of the vestibular dimensions of the follicular spaces depending on the molar position.

**Table 3:**
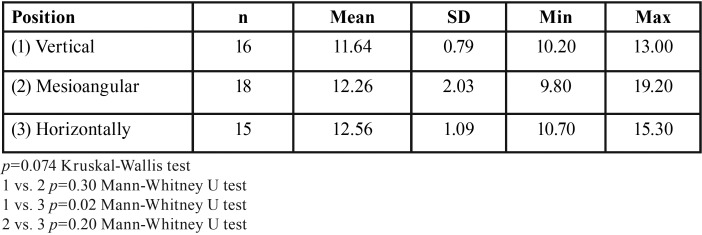
Comparison of the distal dimensions of the follicular spaces depending on the molar position.

**Table 4:**
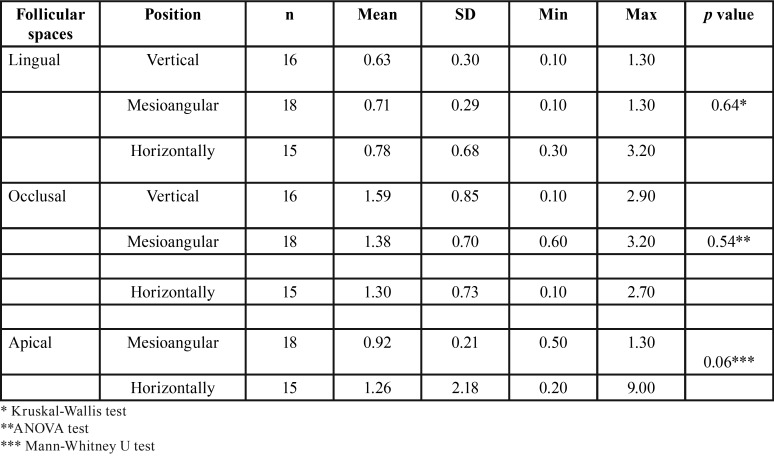
Comparison of the lingual, occlusal and apical dimensions of the follicular spaces depending on the molar position.

## Discussion

The presence of apparent impacted mandibular third molars is a common finding in routine dental examinations (2,3). There is a controversy on whether a small pericoronal radiolucent image may be an indicator of future pathological changes which may support the need for a prophylactic extraction (3,24). There have been studies (12,13) that have investigated impacted mandibular third molars follicles with two-dimensional radiographic imaging and their association with histologically determined findings. Their findings suggested that follicles larger than 2.5 mm could have increased risk for developing associated pathology.

Cone beam computed tomography (CBCT) has been validated in several studies as an accurate and reliable measurement method for craniofacial structures overall. This is at least in part due to the fact that CBCT images consist of isotropic voxels (equal in length, height and length), which enable geometrically accurate measurements in any plane of space (17,25). The impact of this imaging technique on dental treatment planning has also been discussed before (26). In addition, it has been suggested that it is possible to obtain coronal, sagittal, and trans-axial reconstructions in 3D and conventional two-dimensional images from an axial CBCT slice (27). These options make it possible to make a more accurate diagnosis for certain treatment management decisions. To our best knowledge there are no published studies that have quantified FS in mandibular impacted third molars with different angulations using CBCT imaging.

The current results showed statistically significant differences in the vestibular FS dimensions between the third molars groups that were classified as in vertical or mesioangular impacted position. The results also showed statistically significant differences in the mesial FS between the group of third molars in vertical position and those of third molars in mesioangular and horizontal positions. This suggests that mandibular impacted third molars in mesioangular and horizontal positions are more predisposed to develop larger sized FS in certain directions and the likelihood to create a cyst will be increased. Similar results shown that the dimensions of the DF could depend on the mandibular morphology and associated areas of smaller bone resistance. Leitner *et al.* stated that the horizontal position of impacted mandibular third molars tended to be more affected by pathological changes (20). However, Haghanifar *et al.* could not identify a significant association between the DF diameter, the mesiodistal width of the dental crown and the histopathological evaluation (25). It was concluded that the diameter of the DF and the mesiodistal width of the teeth cannot be used as a diagnostic index for differentiating between a normal and pathological DF. It has to be noted that several studies (28,29), recommended prophylactic extraction by other reasons not linked to the DF size, such as the risk of caries and periodontal disease on the adjacent second molar. However, there are not studies to demonstrate the variation of the dental follicle in relation to the angulation of impacted mandibular third molar.

It is suggested to carry out future studies with the aim of establishing reference values of the DF spaces of impacted third molars evaluated through CBCT imaging and their association with an increased likelihood of the presence of (or progression to) a histologically diagnosed cystic or tumor lesion. In cases with a higher pathology development risk prophylactic actions to prevent or decrease the associated adverse effects may be justified.

A reference standard measurement for the FS of normally erupting third molars are lacking. Finally, we refuse the null hypothesis and conclude that the follicles of impacted third molars have different dimensions according to their degree of angulation. Mesioangulated and horizontally positioned IMTMs seems to consistently have some increased FS dimension.

## Conclusions

In this sample IMTM follicles have different dimensions according to their degree of angulation. Mesioangulated and horizontally positioned IMTMs seems to consistently have some increased FS dimensions (mesial and vestibular aspects). No reference standard FS dimensions are available.
